# Notes on Preparations for the Sick

**Published:** 1889-06

**Authors:** 


### Notes on Preparations for the Sick.

---

**Pharmaceutical and Dietetic Preparations.**—H. & T. Kirby & Co., London.

**Glycecols,** or Improved Glycerine Lozenges.—The basis of these lozenges is a compound of glycerine and gelatine, the former in proportion of 50 per cent., without gum or sugar. They are quite unlike the ordinary lozenge; are of jelly-like consistence, lenticular in shape, and weigh from 30 to 40 grains each. They are sweetened with saccharin and medicated in great variety, are both pleasing to the eye and palate, and form a series which should be the most valuable of all forms of lozenge we have met with. The idea of a glycerine lozenge originated with Dr. Kirby, who coined the name Glycecol to represent the colloidal combination of glycerine and gelatine. We have found that they do not contain sugar, inasmuch as they do not reduce Fehling's solution: they are therefore permissible to diabetic patients, who sometimes devour glycerine lozenges, so called, with such avidity that a large excess of sugar appears in the urine in correspondence with that contained in the lozenges taken.

**Piloids.**—This form, available for dry drugs, and also for concentrated tinctures, is a modification of the ordinary tablet, containing compressed drugs and chemicals in definite quantities, in lenticular form, and saving both time and trouble in dispensing. Like pills, they possess the convenience of compactness and portability: they may be swallowed whole as pills, taken as lozenges, reduced to powder, or dissolved in water. The accuracy of dose and readiness for use should give great advantages to the country practitioner in dispensing.

We have now and then found that patients will take medicines in the tablet or piloid form when they will altogether decline to swallow a draught; and, when frequent doses are required, the piloids can be carried in the pocket, and easily taken at regular periods.

A large series of these piloids is prepared, containing a great variety of useful drugs in small doses, which may be taken every hour, or as often as may be desired.

**Soluble Compressed Pellets**, for the immediate production of antiseptic and other lotions and injections.—These pellets, for external use, are put up in coloured bottles; they are intended for vaginal and other injections, two of them being sufficient for a pint of warm water. They are exceedingly convenient, and are a great improvement on all previous methods of dispensing the materials for vaginal and other injections.

**Compressed Beef Tablets.**—These are prepared from beef, condensed with farina. They are about an inch in diameter, and are enveloped in tinfoil. They may be allowed to dissolve in the mouth, or eaten as biscuits. Four of them crushed into powder, and boiled in half-a-pint of water, form a good soup.

**Nutrimentin**, or Condensed Farinated Beef.—This is a new alimentary compound, consisting of fresh meat-powder, whole wheat, oatmeal, and other farinas, forming a nutritious food for invalids. It contains all the meat-substance, except bone and excess of fat; it is, therefore, rich in phosphates and salts, and is, weight for weight, twice as nutritious as fresh meat, being made of dry meat-powder combined with oatmeal. It is largely soluble, and is easily digested by patients in even acute and wasting diseases.

---

**Vapour Cones.**—Chemical Carbon Co., Limited, London.

This ingenious device of the Chemical Carbon Company, for medical and surgical fumigation and disinfection, appears to deserve a cordial reception from the profession, and to be worthy of an extensive investigation.

The material to be volatilised is contained in a small flask, and this flask is surrounded by a hollow cone of pure carbon, combined with an oxidising agent, the whole being mounted on an incombustible base. The glass flask is stoppered with a substance which melts at a low temperature. The cone is used by lighting the top, and the carbonised casing then slowly burns, the flask being gradually heated from above downwards as the carbon burns down. It will be observed that there can be no destruction of the substances to be vaporised, inasmuch as the liquid is rapidly volatilised, and does not boil.

A great variety of these cones are now ready for use, some for general air distribution or direct inhalation, others for the external application of vapours for baths, others for house or chamber disinfection, and others for simple fumigation.

We have tried the ol. pini sylvestris, the camphor-carbolate, and the creasote; and have been much pleased with the way in which they have performed what is expected of them.

They should prove themselves of value in the treatment of respiratory diseases, both for their local action on the mucous membrane and for their germicide powers in bacillary diseases. They must have a wide range of utility, and prove themselves to be superior to all other methods of medicinal inhalation.

---

**Cascara Chocolate Bonbons.**—Ferris & Co., Bristol.

Of the numerous preparations of cascara, this is by far the most pleasing to the eye and palate. Few laxatives have achieved so widely-spread a reputation, and few have established themselves on so secure a footing as the cascara sagrada. The bitter taste has been the one drawback; but an almost tasteless extract is now a possibility, and this in combination with the finest chocolate gives a product in which the taste of the cascara is almost entirely concealed. Each tablet contains a dose equivalent to twenty minims of the fluid extract, and forms quite a luxurious method of administration of a disagreeable but efficient stimulant to peristaltic action.

---

**Boldo-Verne.**—Thos. Christy & Co., London.

Boldo was imported into France ten years ago, and has been shown by experiment to be "the antidote to liver complaints, as quinine is that of fevers." Such statements are easy to make, but more difficult to establish by satisfactory proof. We find that it is said to give especially satisfactory results in the convalescence from typhoid fever; but we fail to see any evidence that the patients would not have recovered without it. "Its action upon the liver, known for such a long time in Chili, has been confirmed in France in many cases at Vichy and Paris principally by Prof. Gubler, who has obtained some remarkable results in congestion and functionary troubles of the liver, hepatic colic, biliary lithiasis, chronic hepatitis, bilious affections, cachexias caused by a long sojourn in warm countries." We shall look for the experience of those who are able to ascertain the effects of the drug in a large class of diseases for which new remedies are very desirable.

Thirty to sixty drops of the boldo-verne are taken in water, wine, coffee, or upon a piece of sugar, twice a day for four days. What could be more simple?

